# Isolation and Genome Annotation of TicTac, an EK1 Cluster Bacteriophage with Podovirus Morphology

**DOI:** 10.17912/micropub.biology.001876

**Published:** 2026-03-27

**Authors:** Megan Allgood, Eva Githuku, Ryan Bonson, Isabella Edwin, Amanda Granander, Nate Kopp, Marcus Kunyu, Ashton Mathews, Precious Ngandu, Austin Pepin, KC Serve, Katelyn Widman, Emily Zelenka, Christopher Zuck, Anna Strimaitis Grinath, Treyton Harris, Jack Shurley, Michael Thomas

**Affiliations:** 1 Biology, Idaho State University, Pocatello, ID, US

## Abstract

TicTac was discovered in the soil underneath a mint bush in Pocatello, Idaho, USA. The bacteriophage has a podovirus morphology, infects *Microbacterium foliorum* NRRL B-24224 and is grouped with bacteriophages in cluster EK1 based on sequence similarity. The genome size is 53,876 bp with 59.0% GC content. Annotation of the genome provided evidence for 57 predicted protein-coding genes, of which 18 have predicted functions, including multiple minor tail proteins.

**Figure 1. Characterization of TicTac:** A) Plaque formation on *M. foliorum* PYCa top agar overlays resulting in small clear plaques. B) Podovirus morphology of TicTac with a capsid diameter of approximately 57 nm and a tail length of approximately 14 nm (n=1). Image produced by a Zeiss EM900 TEM with an accelerating voltage of 80 kV and 1% uranyl acetate negative staining. &nbsp;Electron microscopy performed in ISU’s Molecular Research Core Facility.

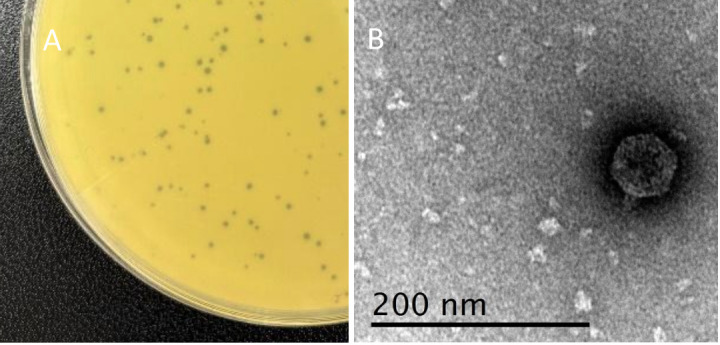



## Description

Bacteriophages are the most abundant microorganism but are largely under-sampled (Batinovic et al., 2019). Despite this, great advances have been made in phage therapy, especially with respect to efforts to combat antimicrobial resistance (Ali et al., 2023; Brives and Pourraz 2020; Gordillo 2019). Here, we describe the isolation and genetic composition of TicTac, a bacteriophage that infects *Microbacterium foliorum* .

TicTac was isolated from a soil sample collected underneath a mint bush in Pocatello, Idaho (42.9 N, 112.39 W) in Fall 2024, following established procedures (Poxleitner et al., 2018). The soil sample was mixed with PYCa liquid media and filtered with a 0.22-μm filter. The filtrate was inoculated with *M. foliorum* NRRL B-24224. After incubation with shaking at 30°C for 48 h, the culture was filtered and the filtrate was plated using *M. foliorum* PYCa top agar overlays, resulting in visible plaques after incubation at 30°C for 48 h. Plaques were purified through three additional rounds of plating ( Fig. 1A ). A high titer lysate was prepared for DNA sequencing and for transmission electron microscopy (TEM). TEM revealed phage of podovirus morphology with short non-contractile tails and icosahedral capsids ( Fig. 1B ). The lack of identifiable tape measure functions in the genome, following genome annotation that is described below, supports the classification of this phage as a podovirus.

Promega Wizard DNA clean up kit was used to extract genomic DNA from a high-titer lysate. Sequencing was performed at the University of Pittsburgh Department of Biological Sciences. A sequencing library was prepared using a NEB Ultra II FS kit. Raw reads were sequenced using a shotgun sequencing approach on an Illumina MiSeq 1000 (v3 reagents), resulting in single-end 150-bp reads with 2,094-fold coverage. Raw reads were trimmed and assembled with Newbler v2.9 using default parameters, yielding a single contig. Consend v29 was used to check for completeness and accuracy and determine phage termini, using default settings (Chung et al., 2017). TicTac’s genome length is 53,876 bp; it is circularly permuted and has a GC content of 59.0%.&nbsp;

The TicTac genome was auto-annotated in DNAmaster v5.23.6 (http://cobamide2.bio.pitt.edu) using Genemark v2.5 (Besemer and Borodovsky 2025) and Glimmer v3.0 (Delcher et al., 2008) and subsequently manual inspected and refined using PECAAN ( http://discover.kbrinsgd.org ) to aggregate results from the following tools: Phamerator v454 (Cresawn et al., 2011) was used for synteny analysis. Starterator (http://phages.wustl.edu/starterator/) was used to identify start sites by manually comparing to other EK1 phages such as phage Birdfeeder (Adams et al., 2022). For rare situations in which Starterator was uninformative or in disagreement with GeneMark and/or Glimmer (5 of 57 genes), we selected the start site that was most commonly used by other EK1 phages, minimized the gap separating the gene from the next upstream gene (optimally, with an overlap of -1 or -4 bp), used the preferred start (AUG codon usage is 99% for Met), and had the highest z-value (measuring the strength of the adjacent Ribosome Binding Site)&nbsp;–&nbsp;see gp30 for an example. HHPred v2.0 (Soding et al., 2005) and BLAST v2.13.0 (Altschul et al., 1990) were used to predict putative gene functions (cutoff values were 10 ^-7^ for BLAST and probability >90% over >50% coverage for HHPred). DeepTMHMM (Hallgren et al., 2022) was used to predict membrane-binding domains, and AlphaFold (Jumper et al. 2021) was used to identify putative function of minor tail proteins. No tRNAs were identified using Aragorn v1.2.41 (Laslett 2004) and tRNA-SE v2.0 (Lowe and Eddy 1997). Using default parameters, the annotation process resulted in the identification of 57 putative protein-coding genes, 18 of which could be assigned gene functions. No immunity repressor or integrase functions could be identified, suggesting TicTac is unlikely to be a temperate phage. TicTac was assigned to cluster EK1 based on gene content similarity of at least 35% to phages in the Actinobacteriophage database, phagesdb ( https://phageDB.org ) (Pope et al., 2017; Russell and Hatfull 2017). Genome annotation subsequently underwent peer review by an experienced SEA-PHAGES-associated faculty and passed QC inspection before submission to GenBank.

Interestingly, the TicTac’s genome contains two notable features. First, a 13461 bp gene of unknown function, gp33, that accounts for 25% of TicTac’s genome. In a tailed phage, this might be suspected of serving as a tape measure protein, though gp33 has no sequence similarity to any known tape measure protein. In a phage with podovirus morphology, like TicTac, the function remains elusive. Second, TicTac’s genome contains several genes that appear to be minor tail proteins (gp 37, 38, 46), key structural components responsible for facilitating host recognition, tail assembly, and genome injection (Hardies et al., 2016; Freeman et al., 2025). This suggests that TicTac may offer insightful information on the diversity of phage tail structure and function within the EK1 cluster of podoviruses. These distinct genomic characteristics render TicTac a promising model for investigations into podovirus structural and functional diversity.

**Data Availability**

Sequence data was deposited in sequence read archive SRR33718578 and GenBank accession number PV876930.

## References

[R1] Adams Benjamin M., Adams Joseph B., Brewster Reganne L., Cutler Michael S., Davis Anthony E., Gallegos Abby H., Hernandez Jeremy S., May Luke H., Montoya Emma G., Reagan Andrew T., Shurley Jack F., Grinath Anna S., Thomas Michael A. (2022). Annotation of the Complete Genome Sequences of Bacteriophages Sara and Birdfeeder. Microbiology Resource Announcements.

[R2] Ali Y, Inusa I, Sanghvi G, Mandaliya VB, Bishoyi AK (2023). The current status of phage therapy and its advancement towards establishing standard antimicrobials for combating multi drug-resistant bacterial pathogens.. Microb Pathog.

[R3] Altschul SF, Gish W, Miller W, Myers EW, Lipman DJ (1990). Basic local alignment search tool.. J Mol Biol.

[R4] Batinovic S, Wassef F, Knowler SA, Rice DTF, Stanton CR, Rose J, Tucci J, Nittami T, Vinh A, Drummond GR, Sobey CG, Chan HT, Seviour RJ, Petrovski S, Franks AE (2019). Bacteriophages in Natural and Artificial Environments.. Pathogens.

[R5] Besemer J, Borodovsky M (2005). GeneMark: web software for gene finding in prokaryotes, eukaryotes and viruses.. Nucleic Acids Res.

[R6] Brives Charlotte, Pourraz Jessica (2020). Phage therapy as a potential solution in the fight against AMR: obstacles and possible futures. Palgrave Communications.

[R7] Chung CH, Walter MH, Yang L, Chen SG, Winston V, Thomas MA (2017). Predicting genome terminus sequences of Bacillus cereus-group bacteriophage using next generation sequencing data.. BMC Genomics.

[R8] Cresawn SG, Bogel M, Day N, Jacobs-Sera D, Hendrix RW, Hatfull GF (2011). Phamerator: a bioinformatic tool for comparative bacteriophage genomics.. BMC Bioinformatics.

[R9] Delcher AL, Bratke KA, Powers EC, Salzberg SL (2007). Identifying bacterial genes and endosymbiont DNA with Glimmer.. Bioinformatics.

[R10] Freeman KG, Mondal S, Macale LS, Podgorski J, White SJ, Silva BH, Ortiz V, Huet A, Perez RJ, Narsico JT, Ho MC, Jacobs-Sera D, Lowary TL, Conway JF, Park D, Hatfull GF (2025). Structure and infection dynamics of mycobacteriophage Bxb1.. Cell.

[R11] Gordillo Altamirano FL, Barr JJ (2019). Phage Therapy in the Postantibiotic Era.. Clin Microbiol Rev.

[R12] Hallgren Jeppe, Tsirigos Konstantinos D., Pedersen Mads Damgaard, Almagro Armenteros José Juan, Marcatili Paolo, Nielsen Henrik, Krogh Anders, Winther Ole (2022). DeepTMHMM predicts alpha and beta transmembrane proteins using deep neural networks.

[R13] Hardies SC, Thomas JA, Black L, Weintraub ST, Hwang CY, Cho BC (2015). Identification of structural and morphogenesis genes of Pseudoalteromonas phage φRIO-1 and placement within the evolutionary history of Podoviridae.. Virology.

[R14] Jumper J, Evans R, Pritzel A, Green T, Figurnov M, Ronneberger O, Tunyasuvunakool K, Bates R, Žídek A, Potapenko A, Bridgland A, Meyer C, Kohl SAA, Ballard AJ, Cowie A, Romera-Paredes B, Nikolov S, Jain R, Adler J, Back T, Petersen S, Reiman D, Clancy E, Zielinski M, Steinegger M, Pacholska M, Berghammer T, Bodenstein S, Silver D, Vinyals O, Senior AW, Kavukcuoglu K, Kohli P, Hassabis D (2021). Highly accurate protein structure prediction with AlphaFold.. Nature.

[R15] Laslett D, Canback B (2004). ARAGORN, a program to detect tRNA genes and tmRNA genes in nucleotide sequences.. Nucleic Acids Res.

[R16] Lowe TM, Eddy SR (1997). tRNAscan-SE: a program for improved detection of transfer RNA genes in genomic sequence.. Nucleic Acids Res.

[R17] Pope WH, Mavrich TN, Garlena RA, Guerrero-Bustamante CA, Jacobs-Sera D, Montgomery MT, Russell DA, Warner MH, Hatfull GF, Science Education Alliance-Phage Hunters Advancing Genomics and Evolutionary Science (SEA-PHAGES) (2017). Bacteriophages of Gordonia spp. Display a Spectrum of Diversity and Genetic Relationships.. mBio.

[R18] Poxleitner M, Pope W, Jacobs-Sera D, Sivanathan V, and Hatfull G. 2018. Phage discovery guide. Howard Hughes Medical Institute, Chevy Chase, MD.

[R19] Russell DA, Hatfull GF (2017). PhagesDB: the actinobacteriophage database.. Bioinformatics.

[R20] Söding J, Biegert A, Lupas AN (2005). The HHpred interactive server for protein homology detection and structure prediction.. Nucleic Acids Res.

